# Challenges of competency-based curriculum in teaching learners with learning disabilities

**DOI:** 10.4102/ajod.v13i0.1268

**Published:** 2024-03-04

**Authors:** Jabulani Mpofu, Maximus M. Sefotho

**Affiliations:** 1 Department of Educational Psychology, Faculty of Education, University of Johannesburg, Johannesburg, South Africa; 2 Department of Disability Studies, Faculty of Applied Social Sciences, Zimbabwe Open University, Harare, Zimbabwe; 3 Division of Disability Studies, Department of Health and Rehabilitation Sciences, Faculty of Health Sciences, University of Cape Town, Cape Town, South Africa

**Keywords:** attitudes, challenges, competency-based curriculum, learners with learning disabilities, teachers

## Abstract

**Background:**

Zimbabwean government adopted competency-based curriculum in 2017 as a measure to prepare learners for life and work in an indigenised economy and increasingly globalised and competitive environment. The government also sought to ensure that learners develop skills necessary for lifelong learning in line with the emerging opportunities.

**Objectives:**

The purpose of this study was to explore challenges faced by teachers in the implementation of competency-based curriculum to learners with learning disabilities in Mhangura of Makonde District in Zimbabwe.

**Methods:**

A constructivist lived experience perspective underpinned this research, in which a single case study was used to interact with participants on challenges faced by teachers in the implementation of competency-based curriculum to learners with learning disabilities. Purposive sampling was used to select nine participants (five males and four females). Data were collected through face-to-face interviews and transcribed verbatim. Four themes emerged from the thematic analysis of data sources.

**Results:**

Results indicated that participants were facing several challenges in implementing competency-based curriculum to learners with learning disabilities. Among the cited challenges were negative attitudes towards learners with learning disabilities, poor teacher preparation, lack of resources and poor collaboration.

**Conclusion:**

The study concluded that the objectives of competency-based curriculum are noble to learners, but its implementation is not inclusive.

**Contribution:**

The study findings will assist in identifying areas that need to be improved and need strengthening. The education policy makers in the country will have a better understanding of challenges faced by teachers in the implementation of competency-based curriculum in Zimbabwe.

## Introduction

Competency-based curriculum is a type of learning that focuses on acquisition of skills, abilities, personal traits, capacities, knowledge and values (Boahin 2018). It is different from traditional curriculum where learning is based on academic framework where achievement is judged by the ability to recall key points, information imparted or sequences memorised. Competency-based curriculum focuses on what a student knows and can do rather than on how the student learns. It measures learning rather than time. Competency-based curriculum progresses by allowing learners to demonstrate their competencies (Mkonongwa 2019). This means learners have to prove that they mastered the knowledge and skills required for a particular course regardless of how long it takes and they should be able to apply the skills in various life situations. It helps learners prepare for life and work by ensuring that they are equipped with the requisite knowledge, skill and attitude (Mulenga 2018). In a competency-based curriculum, there is inclusion of active learning; this helps to produce learners who are holistic, creative, innovative, analytical and cooperative in their communities and nation. Bratianu, Hadad and Bejinaru (2020) posit that competence-based curriculum requires a paradigm shift from assessing learning content to assessing learning outcomes. Competency-based curriculum looks at life after school and the outcomes of educational systems, prepares and orients learners for participation in voluntary services leadership (Manokore 2022). Competency-based curriculum fosters lifelong learning in line with the emerging opportunities and challenges of the society. It focuses on what learners are expected to do rather than on what they are expected to know. It is a learner-centred curriculum that is adaptive to the changing needs of students, teachers and society instead of focusing on grades and yearly curriculum schedules (Boahin 2018). The main focus is placed on how competent each student is in the subject by demonstrating mastery. Competency-based curriculum and personalised learning go hand in hand. It prepares children for the next stage of their life and is for all learners including those with learning disabilities (Ankam et al. 2019).

Competency-based curriculum was developed as an educational trend in the United States. Canada is one of the countries that implemented the competency-based curriculum. The goal of competency-based curriculum in Canada was to reduce the existing gap between what is prescribed in programmes of study and what really happens in the classroom (Griffith 2021). A review of competency-based curriculum to evaluate its successes on general basic adult education, through analysis of actions of students in context as well as the resources was carried in Canada. The evaluation found out that competency-based learning needed a lot of resources to address everyday problems that were not traditionally associated with school subjects. China also adopted competency-based curriculum in 2005 aiming to cultivate student competencies in practical situations in primary and secondary education (Fein 2015). Review of the Chinese competency-based curriculum found out that it was more political than pedagogical. Guatemala has been implementing a competency-based curriculum from preschool to secondary education. The Guatemala competency-based curriculum is embedded in the ongoing expansion and democratisation of learning conditions and opportunities. A review of Guatemala competency-based curriculum also indicated that it needed a lot of resources and it was time consuming.

Most African countries (e.g. Tanzania, Rwanda, Kenya, Zimbabwe) have recently incorporated competency-based curriculum into their education system. However, it has not been widely disseminated or implemented in many classrooms because of the varied educational challenges affecting African countries (UNESCO 2015). In 2014, the Zimbabwean Ministry of Primary and Secondary Education embarked on a comprehensive curriculum reform process meant to enhance the quality of education in Zimbabwe (Manokore 2022). It adopted competency-based curriculum in 2017 as a vehicle to motivate learners to cherish their Zimbabwean heritage, history and cultural traditions and prepare them for participatory citizenship (Manokore 2022). The Zimbabwean government is yet to make a review of its competency-based curriculum.

Zimbabwe has signed the United Nation’s Convention on the Rights of Persons with Disabilities (UNCRPD) and, therefore, has legally adopted the UN definition of learners with a learning disability. The United Nations defines learning disability as a neurodevelopmental disorder that affects an individual’s ability to acquire, process and retain academic skills despite average or above-average intelligence and sociocultural opportunities (American Psychiatric Association 2013). It is characterised by significant and persistent difficulties in one or more areas of learning, such as reading, writing, mathematics or comprehension. These difficulties stem from a variety of underlying factors, including but not limited to, cognitive processing deficits, attentional difficulties and language impairments (Fletcher et al. 2019). The impact of learning disabilities can vary widely, with each individual presenting unique challenges and strengths. Early identification, appropriate interventions and accommodations are crucial in ensuring that individuals with learning disabilities receive the necessary support to succeed academically and reach their full potential (Fletcher et al. 2019). Learners with learning disabilities should be educated in general education classrooms and learn the same content with their peers (Hove & Phasha 2023). Assessment of learners with disabilities usually takes a multidimensional perspective. Multidimensional perspective in assessment of learning disabilities takes into account the primary factors contributing to the child’s learning problems as well as interaction of these influences on learning. In other words, the assumption is that learning disabilities may result in neuropsychological, developmental and behavioural factors. An in-depth assessment of all contributing factors is necessary for effective interventions. Teachers should be in a position to modify teaching approaches (Haleem et al. 2022) and an enabling environment that is welcome has to be created within the education system. Teachers must be equipped with relevant and effective teaching skills of the inclusive classes. To cater for unique needs of the learners with learning disabilities, the individual educational plan (IEP) is an important tool (Allison & Robison-Young 2016). In the IEP, long- and short-term goals suitable for the learner are set. If there is no progress made, the IEP is modified or discarded to come up with a realistic goal.

## Statement of the problem

Competency-based curriculum is a type of learning that is aimed at ensuring that students acquire knowledge and skills that are deemed to be essential to success in school, careers and adult life. It is a system of instruction, assessment, grading and academic reporting based on students demonstrating that they have learned the knowledge and skills they are expected to learn as they progress through their education. In Zimbabwe, competency-based curriculum was developed in 2014 and the implementation commenced in 2017. Zimbabwean teachers are implementing competency-based curriculum. Competency-based curriculum is giving a lot of challenges to Zimbabwean teachers, and since its inception, no evaluation on its success and challenges has been done by the Zimbabwean Ministry of Primary and Secondary Education. There is also little achievement in the skills development in mainstream schools in Zimbabwe (Manokore 2022). This study explored challenges faced by teachers in the implementation of competency-based curriculum for learners with learning disabilities in Mhangura of Makonde District in Zimbabwe.

## Research methods and design

This article used a qualitative method for exploration of challenges faced by teachers in the implementation of competency-based curriculum for learners with learning disabilities in Zimbabwe using a variety of data sources (Creswell 2014; Eatough & Finlay 2012). The qualitative research approach ensured that exploration of challenges faced by teachers in the implementation of competency-based curriculum for learners with learning disabilities is not examined through a single lens, but rather through different lenses, which allows rich data to be collected on aspects of the phenomenon to be revealed and understood (Castillo-Montoya 2016; Creswell 2012). A qualitative research method was chosen because it allowed the researchers to provide a rich and vivid description of challenges faced by teachers in the implementation of competency-based curriculum for learners with learning disabilities. The qualitative research approach design also enabled the study participants to air their views on experiences and voice challenges faced by teachers in the implementation of competency-based curriculum for learners with learning disabilities (Chowdhury 2015). Information obtained from this study was treated as the real experiences of the participants; therefore, relevant conclusions were drawn about the phenomenon under study.

### Research design

The case study design was preferred for its ‘…ability to investigate complex social phenomena and to handle dense data’ (Glette & Wiig 2022). The study used a single case study as its research design (Leelarungrayub et al. 2020). A single case design is differentiated from a multiple case study in that multiple case study designs study two or more cases (Yin 2018). A single case study was relevant to this study. A case study analyses a single unit – in this study, competency-based curriculum in teaching learners with learning disabilities. Individual interviews are a valuable research method within the framework of single case studies because of their ability to provide in-depth insights into a particular phenomenon (Yin 2014). In this study, a single case study enabled the researchers to obtain a detailed exploration of challenges faced by teachers in the implementation of competency-based curriculum to learners with learning disabilities in real-life settings (Ruth & Ramadas 2019). In the context of this study, individual interviews and focus group discussions were conducted with multiple participants, who possess unique perspectives and experiences related to the challenges faced by teachers in the implementation of competency-based curriculum for learners with learning disabilities (Elliott & Timulak 2015). By conducting multiple interviews and focus group discussions, the researchers gathered rich and detailed data about each individual’s perceptions, thoughts and experiences, allowing for a more comprehensive understanding of the challenges faced by teachers in the implementation of competency-based curriculum for learners with learning disabilities. This study used only one data source and this limits the depth and breath of analysis, leading to potential bias.

### Setting

Participants were drawn from Mhangura in Makonde District, which is the provincial capital of the Mashonaland West Province in Zimbabwe. The study was specifically undertaken in Mhangura of Makonde District because it is one of the districts that pioneered the implementation of competency-based curriculum in Zimbabwe. Mhangura is predominantly a farming area with an estimated population of 1000 teachers. The collapse of industries and poor performance of the agrarian sector in Zimbabwe contributed to poor social services for learners with disabilities as the country is running on a stringent budget (Mpofu 2023). Institutionalised social services for learners with learning disabilities such as special schools and resource centres were closed as they demanded huge resources from the national budget (Mpofu 2023). The government could not sustain their costly overheads (e.g. lower teacher-student ratios, specialist counsellors, teachers and equipment) and hence opted to involve communities to manage the needs of some of its people with disabilities (Mpofu & Shumba 2012). Communities such as Mhangura were called in to take care of those with mild to moderate disabilities (Mpofu 2021). The Zimbabwean government encouraged these communities to be inclusive and designed never-ending strategies to effectively address diversity in these communities by identifying and removing barriers to community adjustment, development and participation (Mpofu 2021).

### Study sample and sampling strategy

The authors purposefully sampled nine (five females, four males) participants to participate in this study. This study included participants who were teachers teaching classes with learners diagnosed with learning disabilities but learning in mainstream classes in Mhangura of Makonde District, Mashonaland West Province in Zimbabwe. This sample was drawn from teachers who were pioneers in the implementation of competency-based curriculum in the country. To be included in the study, the teachers were supposed to have learners with learning disabilities in their classes for an intermittent period of 2 years. The sample was also adequate for a study that used interviews as its data collection tool. The sample also enabled the researchers to achieve data saturation (Creswell 2012, 2014).

Table 1 provides additional demographic details. The names in the table are pseudonyms used for the purpose of this study only.

**TABLE 1:** Demographic information of research participants.

Serial	Gender	Pseudonyms	Years in service	Training in CBC
1	Male	Paul	17	No
2	Female	Patience	4	No
3	Female	Nyarai	15	No
4	Female	Tracy	10	No
5	Male	John	5	No
6	Male	Gift	1	No
7	Male	Ebenezer	7	No
8	Female	Beverly	9	No
9	Female	Charity	6	No

CBC, competency-based curriculum.

### Data collection

Consistent with the qualitative research methodology, this study adopted the use of open-ended interviews and focus group discussions to collect data (Elliott & Timulak 2015). Participants responded to one-on-one interview questions based on a prepared interview schedule on how teachers perceive challenges of the implementation of competency-based curriculum to learners with learning disabilities. The same participants were also engaged in two focus group discussions where they discussed their perceptions on challenges of the implementation of competency-based curriculum to learners with learning disabilities. This approach specifically aimed to facilitate accessing experiences and voices of teachers’ challenges in implementation of competency-based curriculum to learners with learning disabilities in Mhangura of Makonde District in Zimbabwe (Elliott & Timulak 2015). The interviews were recorded using a mobile phone, with each interview lasting between 60 and 90 min. The interviews were conducted, transcribed and analysed by the first author.

### Data analysis strategies

Data analysis was done using the thematic content analysis approach (Etikan et al. 2016). The first stage involved becoming familiar with the data. This was done through listening to and transcribing of the interviews. The second stage involved creating codes linked to research goals by identifying keywords and sentences (Etikan et al. 2016). The third stage involved grouping codes into themes and the last stage involved reviewing themes, labelling them and selecting appropriate quotes to represent the themes identified from each transcript. In carrying out the analysis, coding was not only data driven but also influenced by the study’s aim (Etikan et al. 2016).

### Rigour of the study

To ensure rigour of this study, we checked for credibility, dependability, confirmability and transferability (Fusch & Ness 2015). To enhance the credibility of our study, we prolonged the engagement of our participants through engaging them in interviews that lasted more than an hour. We also triangulated the nine interviews held and two focus group discussions to produce a more comprehensive view of the phenomenon being studied. We also allowed peer debriefing in this study in order to see agreement in data labels and the logical paths taken to arrive at those labels. We also allowed member checking in this study (Fusch & Ness 2015). We allowed participants to read the transcription of their interviews and focus groups to ensure that these had been accurately recorded and were therefore credible (Guest, MacQueen & Namey 2012). In addition to credibility checking, we also checked for the study’s dependability and confirmability by making an audit trail to our study and authors’ reflexivity. We also provided thick description throughout our study to check for the transferability of our study.

### Ethical considerations

Ethical approval for the study was obtained from the Ethics Committee of the Zimbabwe Open University. Ethical Review Board of the Zimbabwe Open University provided ethical clearance prior to commencing the study: #6001/23. Standard ethical principles of informed consent and voluntary participation, protection from harm, confidentiality and privacy, were adhered to throughout the research process and data collection and analysis. Assurance was given that no person would be identified.

### Findings

The findings of this study are divided into four main themes:

1. Negative attitudes towards learners with learning disabilities.
2. Poor teacher preparation.
3. Inadequate resources.
4. Stakeholder collaboration.

### Negative attitudes towards learners with learning disabilities

Most participants (*n* = 7) in this study reported that negative attitudes from both teachers and community members towards learners with learning disabilities were contributing to challenges in effective implementation of competency-based curriculum for the benefit of learners in their classes. The following are verbatims obtained from the study interviews: female (f), years in service (y), not trained in competency-based curriculum (NTCBN).

Tracy stated:

‘… also negative attitude of the community and teachers hinders the implementation of Competency-Based Curriculum at this school. Parents have negative attitude towards learners with disabilities, they think it’s a waste of money and resources to invest in their education. They just bring their learners, dump them here, they don’t pay fees, they don’t buy exercise books. With all this it becomes difficult to implement Competency-Based Curriculum.’ (f, 10 y, NTCBM)

Gift added:

‘To start with let me say parents have developed negative attitude towards Competency-Based Curriculum. They look down upon it saying it has made education expensive than before, especially with the issue of Continuous Assessment Learning Activity (CALA). Parents are not contributing much resources, this then makes the implementation of Competency-Based Curriculum difficult.’ (m, 1 y, NTCBM)

Beverly also submitted that:

‘Discrimination and stigmatisation from teachers, other learners without disabilities and community’s negative attitude towards learners with learning disabilities is one of the barriers to successful implementation of Competency-Based Curriculum. Let me say because of lack of knowledge that disability is not inability teachers, learners and community at large are not prepared to accept learners with disabilities. Until society attitude towards learners with disabilities changes Competency-Based Curriculum will remain difficult to implement for learners with learning disabilities.’ (f, 9 y, NTCBM)

### Poor teacher preparation

Participants from this study (*n* = 7) also indicated that they were not trained to implement competency-based curriculum, and this was a great challenge to their implementation of the curriculum and putting more risks to learners with learning disabilities.

Paul has this to say:

‘The problem is most of the teachers here if not all of them lack in service training in Competency-Based Curriculum hence most are still using the old teaching approach. In service enhances skills of how to implement Competency-Based Curriculum for learners with disabilities. Without relevant skills teaching is hampered and would not be meaningful. We really want to implement Competency-Based Curriculum but we lack the pedagogy.’ (m, 17 y, NTCBM)

Patience also added:

‘A number of teachers here are still using the old teaching approach and same teaching materials from the previous year’s instead of adopting Competency-Based Curriculum teaching methods. We were not trained in Competency-Based Curriculum hence we lack methodologies. Competency-Based Curriculum is tiresome in terms in terms of materials and this affect effort towards its implementation.’ (f, 4 y, NTCBM)

And John submitted:

‘… also, teachers here did not receive training in Competency-Based Curriculum. This limit their pedagogical knowledge and skills to implement Competency-Based Curriculum. You know with lack of knowledge teachers fail to deal with complex learning problems such as those of learners with disabilities.’ (f, 5 y, NTCBM)

Gift narrated:

‘Competency-Based Curriculum to learners with disabilities still remains a realm of theory and far from practice at this school. Teachers were not trained in Competency-Based Curriculum don’t know how to handle learners with disabilities and teaching them becomes a challenge. Competency-Based Curriculum is overloaded. And this have negative consequences for both the teacher and the learners.’ (m, 1 y, NTCBM)

Ebenezer alluded that:

‘The other issue is we were not trained in Competency-Based Curriculum. Even our administrators were not. They are not knowledgeable about the practices and facilities that need to be made available to implement Competency-Based Curriculum. They lack even the assessment rubrics and this have impact on its implementation. Teachers also lack understanding of Competency-Based Curriculum this forces teachers to ignore it. If you move around and look at their schemes of work, you will see that they do not reflect the qualities of Competency-Based Curriculum.’ (m, 7y, NTCBM)

Beverly submitted that:

‘We do not have specialist teachers to help us in handling learners with disabilities in the implementation of Competency-Based Curriculum School.’ (m, 9 y, NTCBM)

And Charity also:

‘It is a challenge implementing Competency-Based Curriculum especially to learners with disabilities. We do not have specialist teachers to guide us in teaching learners with learning disabilities. We lack knowledge on how to handle learners with learning disabilities. Competency-Based Curriculum requires a unique set of competencies such as knowledge and skills of teaching strategies that meet the needs of learners with learning disabilities. Its unfortunate; we were never equipped with them.’ (f, 6 y, NTCBM)

### Lack of resources

Most participants (*n* = 5) who responded to the interview indicated that they were lacking resources to adequately meet and successfully implement competency-based curriculum especially when it comes to learners with disabilities in their mainstream classes.

Paul added:

‘… also, unavailability of appropriate teaching and learning materials hinder the implementation of Competency-Based Curriculum. At this school we do not have enough textbooks to satisfy the increased number of students at this school. Book student ratio is 1:10. You can imagine how challenging the situation can be. Just imagine learners with learning disabilities sharing one book. Learning can never be effective. To add on the issues of resources we don’t have laboratories where we conduct practical lessons. Learners just learn theories and principles yet Competency-Based Curriculum is fully packed with experiments.’ (m, 17 y, NTCBM)

Patience said:

‘On the issue of resources, we do not have computer labs, photocopiers just to mention a few. It then becomes difficult to implement it. The admin is failing to buy chalks what more of textbooks. Learners with disabilities need time with their teachers but here is like most teachers have more than 40 learners in class. I have 60 learners 38 for grade 4 and 22 for grade 4, it is a composite class. This then becomes a challenge in implementing Competency-Based Curriculum.’ (f, 4 y, NTCBM)

She went on to add:

‘… our classrooms are not enough and we do not have adequate textbooks. In some classes there is only one text book per subject and at primary level this is just a mockery especially looking at the requirements of Competency-Based Curriculum. Our school is poor; we sometimes fail to buy chalks. Parents are failing to play their role because of poverty. They cannot combine their resources with that of the school to promote the implementation of Competency-Based Curriculum.’ (f, 4 y, NTCBM)

John added:

‘The greatest challenge here is that of large classes. Teacher learner interaction is hindered and learner to learner interaction do not take place effectively. With higher density classes the teacher fails to know the learning styles of learners especially those with disabilities. The classes are too big for the implementation of Competency-Based Curriculum. It actually means the teacher has limited time to attend to all the learners. Resources are a challenge in implementing Competency-Based Curriculum. Due to lack of funding the school fails to buy simple things like pens for teachers. This makes the implementation of Competency-Based Curriculum difficult.’ (m, 5 y, NTCBM)

Ebenezer contributed by saying:

‘The government is not providing us with the resources to effectively implement Competency-Based Curriculum for learners with disabilities. The ministry of education wants us to implement Competency-Based Curriculum and where to get the resources is it’s our baby to nurse. Learners are not paying fees and there is no way we can force them to pay. We do not have enough classrooms where learning can take place effectively. Most of the time learning. takes place outside the classroom, we do not have laboratories to carry out experiments.’ (m, 7 y, NTCBM)

### Stakeholder collaboration

The study participants also cited a lack of collaboration between major stakeholders in teaching and learning of learners with learning disabilities in their mainstream classes. The cited stakeholders were the government and schools’ psychological services, parents and teachers.

Paul said:

‘We do not have functional psychological and welfare services. We only hear that there are school psychologists but we have not seen them here. Learners with disabilities need counselling here and there. No psychological and social services officers ever visited this school to offer professional guidance and counselling support services to special needs learners.’ (m, 17 y, NTCBM)

And the following is a verbatim from Patience:

‘Another challenge is that teachers were not much involved in developing Competency-Based Curriculum therefore they are not willing to contribute in things they were not much involved. This then becomes a problem in implementing Competency-Based Curriculum.’ (f, 4 y, NTCBM)

John added:

‘… Lack of parental involvement is another challenge. Parents are much involved in farming to the extent that they do not have time to be involved in school things and they do not understand what Competency-Based Curriculum is all about.’ (m, 5 y, NTCBM)

And Gift contributed by saying:

‘I do everything here I play the role of schools psychological services by assessing the developmental progress of the child. I teach the child and help her do homework as well. This is very tiresome we must work together as stakeholders to help the child. Remember this is not a special class but mainstream class.’ (m, 1 y, NTCBM)

Beverly also said:

‘Lack of parental involvement is putting a lot of us under pressure here, there is need for their support especially on the issue of resources but because they also lack knowledge on Competency-Based Curriculum they have withdrawn giving a helping hand. They are claiming this approach has made education expensive considering their poor economic status, they are unemployed as a result of country’s economic meltdown. There is also shortage of human resources at this school, for example most of my time I spend it on administrative work than the important work I have come here to do.’ (f, 9 y, NTCBM)

Charity added:

‘It is difficult to implement something we were not involved in its crafting, very difficult especially when dealing with learners with disabilities. You know involvement in the initial stages promotes ownership of change and innovation. We were not involved in the initial stage hence we lack intrinsic motivation to implement Competency-Based Curriculum especially to learners with learning disabilities, because it is not an innovative, we designed, it is something which was imposed on us.’ (f, 6 y, NTCBM)

## Discussion of findings

### Negative attitudes

The study found out that the implementation of competency-based curriculum towards teaching of learners with learning disabilities was being affected by the teachers’ negative attitudes towards learners with disabilities. Teachers admitted that together with parents of learners with disabilities, they have negative attitudes towards learners with disabilities and hence it was one of their challenges. Their position suggests that they have religious or cultural beliefs that are negative towards learners with learning disabilities. These beliefs were hindering them in providing competency-based curriculum to learners with learning disabilities in their classes.

Teachers’ attitudes are essential elements of professional competence (Gebhardt et al. 2015). Positive attitudes towards inclusion have a significant role in the implementation of any school curriculum (Gebhardt et al. 2015). A number of researches have indicated that negative attitudes of teachers towards those with disabilities compromise teaching and learning of learners with disabilities. Negative terms that are used by teachers to refer to learners with learning disabilities reflect how teachers perceive and teach learners with disabilities. The terms that are used by wider society to describe persons with learning disabilities tend to be negative. This makes the implementation of competency-based curriculum for learners with learning disabilities in mainstream classes a challenge for teachers (Gebhardt et al. 2015). Although study in Nigeria showed that teachers’ attitude to students with special needs had greatly improved as a result of workshops, seminars and conferences attended by teachers, the study also found out that most teachers still had negative attitudes to students with special needs.

### Poor teacher preparation

All the participants in this study indicated that competency-based curriculum was just imposed on them. They said they were not trained to implement it and were just implementing it from the heads’ feedback from the Ministry of Primary and Secondary Education through announcements and circulars. Their heads were not trained or inducted in competency-based curriculum too.

They said this was presenting them with challenges especially in classes with learners diagnosed with learning disabilities. They explained that they have no competency-based curriculum pedagogy and some are just teaching it like they used to do with the old curriculum.

Lack of training in the implementation of competency-based curriculum was also cited as a major challenge in the implementation of competency-based curriculum in classes with learners with learning disabilities. Idah (2017) conducted a research on the effectiveness of teacher preparation in the implementation of competency-based curriculum using student teachers and found out that teachers who were exposed to competency-based curriculum as part of their training were facing few challenges in the implementation of competency-based curriculum. Such teachers were found to be very patient and respectful to the needs of their learners. A good teacher is a friend, a master and has an excellent subject knowledge (Idah 2017). A good competency-based curriculum teacher makes learners understand the teaching material, objectives, pays attention to the learners and helps learners with their problems while encouraging them. The same researcher (Idah 2017) found out that teachers who are not trained in any form of curriculum regularly check learners’ achievement, consistency, discipline them, keep the school rules, deal with the most talented students, play on the lessons and give a lot of homework without teaching. Therefore, this brings out the importance of training of curriculum before implementation. If implemented, there is a need for in-service training.

### Inadequate resources

Most participants mentioned that they lacked resources in the implementation of competency-based curriculum to learners with learning disabilities. They mentioned that the book student ratio is 1:10, and they do not have physical resources such as laboratories to carry out experiments and that they lack simple provisions such as chalks and this hinders the implementation of competency-based curriculum to learners with learning disabilities who need adequate resources in their learning.

Chinangure and Chindanya (2019) concur that when schools undermine facilities, performance of teachers in teaching learners with learning disabilities suffers. Competency-based curriculum requires education institutions to supply adequate learning resources such as modern classrooms, laboratories and latest technology at all levels. Facilities help teachers to teach effectively, and they are requisites of competency-based curriculum. Without enough facilities, teachers cannot effectively help learners to develop independent learning skills and problem-solving skills. This problem of lack of resources affects students and teachers, which in turn can affect the parents of the children (Maffea 2020). The lack of resources in classrooms can cause extreme distress on the students and teachers. Not only are the students and teachers in distress but also they are unable to learn to their fullest potential because they are not being given the proper resources (Maffea 2020).

The participants also echoed that there were low human resources at their school. Low human resources in terms of teachers make them teach big classes that are not compatible with learning needs of learners with learning disabilities. They said learners with learning disabilities need reasonable teacher-pupil ratio that enables them to interact well with their learners. Some indicated that they teach classes with more than 60 learners, and within these classes, they have learners with learning disabilities. To them, this creates fatigue and mirror teaching, which is not of great benefit to them and the learners too.

The findings are similar to the findings of various different researchers, who state that when a large group of learners are combined in one classroom, teachers do not teach effectively and get fatigued with their job, and this puts learners with learning disabilities at more risk (Chinangure & Chindanya 2019). Bottiani et al. (2019) also noted that even for teachers who are highly skilled and have a myriad of personal resources, decision-making and teaching practices may be hindered by stress and burnout arising from high demands and low organisational resources. Furthermore, these teachers stand in front of a class of around 20–30 students a day and have to deal with the children themselves, the lesson plan and the lack of resources. The job itself already takes a toll on them, and when the stress of teaching in a classroom with not enough resources is added, it can lead the teachers to lose their passion. Once this happens, the students are the ones who suffer the consequences.

### Stakeholder collaboration

The study results also showed that there was no effective collaboration in the implementation of competency-based curriculum in schools. The findings suggest that there is a need for good collaboration between major stakeholders to obtain effective implementation of competency-based curriculum in schools.

Competency-based curriculum in inclusive education setting cannot be implemented successfully without collaboration-based relationships (Avalos-Bevan & Bascope 2017; Hall & Wurf 2018; Zagona, Kurth & Macfarland 2017). For teachers and other stakeholders to successfully implement competency-based curriculum in inclusive education in schools, they need to collaborate (Hall & Wurf 2018; Zagona et al. 2017). Successful execution of competency-based curriculum in inclusive education means stipulation of quality education and assurance that the needs of learners who experience learning barriers are met. Therefore, teachers and other support personnel need to collaborate for the successful implementation of competency-based curriculum (Da Fonte & Barton-Arwood 2017).

Keef and Moore (2004) concur with the above findings by confirming that in order to reduce the burden from teachers in teaching learners with special needs in mainstream class settings so as to meet their needs, there is a need for effective stakeholder collaboration. The authors (Keef & Moore 2004) went on to cite teachers’–parents’ government learner support unit and special teachers as important people in education settings that are inclusive in nature. Gately and Gately (2001) also suggest that collaboration teaching at the elementary level is important. Jackson, Ryndak and Billingsley (2000) note that at the secondary level, collaboration makes teaching enjoyable and learning successful to learners with disabilities in mainstream schools.

### Limitations of the study

This study followed a single case study design. Case studies are known for being subjective, biased, or lacking in rigor. In order to address these limitations, data collection was through interviews and focus group discussions. There was also prolonged engagement with participants. We are aware that case study results are not generalisable, and therefore suggest that results from this study only be applied to cases similar to ours.

## Conclusion

This study concluded that mainstream class teachers with learners with learning disabilities in Mhangura of Makonde District in Zimbabwe were facing challenges in the implementation of competency-based curriculum in their classes, especially when it comes to help learners with learning disabilities. Respondents cited that the negative attitudes they hold towards learners with learning disabilities, poor teacher preparation, lack of teaching and learning resources and weak collaboration from relevant stakeholders are the major challenges in the implementation of competency-based curriculum in their classes.

## Study recommendations

Basing on the study findings, this study recommends the following recommendations: there is a need to provide inclusive education in-service training to Zimbabwean teachers. This inclusive education service training will assist teachers in understanding the learning needs of their diversified learners. The study also recommends adequate resources for smooth implementation of competency-based curriculum in Zimbabwean mainstream schools. There is also a need for effective collaboration in the implementation of competency-based curriculum in Zimbabwean schools. All stakeholders must be involved in the implementation of competency-based curriculum especially where there are learners with special needs.
